# QTLs underlying the genetic interrelationship between efficient compatibility of *Bradyrhizobium* strains with soybean and genistein secretion by soybean roots

**DOI:** 10.1371/journal.pone.0194671

**Published:** 2018-04-04

**Authors:** Clarissien Ramongolalaina, Masayoshi Teraishi, Yutaka Okumoto

**Affiliations:** Graduate School of Agriculture, Kyoto University, Oiwake, Kitashirakawa, Sakyo, Kyoto, Japan; College of Agricultural Sciences, UNITED STATES

## Abstract

Soybean plants establish symbiotic relationships with soil rhizobia which form nodules on the plant roots. Nodule formation starts when the plant roots exudate isoflavonoids that induce *nod* gene expression of a specific *Bradyrhizobium*. We examined the specific indigenous rhizobia that form nodules with the soybean cultivars Peking and Tamahomare in different soils. PCR-RFLP analysis targeted to the 16S-23S rRNA gene internal transcribed spacer (ITS) region of the bacterial type of each root nodule showed that *Bradyrhizobium japonicum* (USDA110-type) and *Bradyrhizobium elkanii* (USDA94-type) had high compatibility with the Tamahomare and Peking cultivars, respectively. We grew 93 recombinant inbred lines (RIL) of soybean seeds derived from the cross between Peking and Tamahomare in three different field soils and identified the indigenous rhizobia nodulating each line using the same PCR-RFLP analysis. QTL analysis identified one QTL region in chromosome-18 with a highly significant additive effect that controls compatibility with both *B*. *japonicum* USDA110 and *B*. *elkanii* USDA94. We also measured the amount of daidzein and genistein secretion from roots of the 93 RILs by HPLC analysis. QTL analysis showed one QTL region in chromosome-18 controlling genistein secretion from roots and coinciding with that regulating compatibility of specific indigenous rhizobia with soybean. The amount of genistein may be a major regulatory factor in soybean-rhizobium compatibility.

## Introduction

Soybean (*Glycine max*) is one of the most important crops in the world. It is highly suitable for human and animal diets and is the source of 30% of the world’s oil derived from processed crops. From 2010 to 2014, its planted area increased from 111 to 124 million ha and its production quantity increased from 280 to 320 million tons. The current largest producers are the USA (34%), Brazil (27%) and Argentina (17%) (FAO, 2017). The use of legumes in biofuel production will further increase the economic impact of soybean[1,2]. Its rising economic importance has led to increased efforts over the past several years to improve soybean productivity. Enhancing the efficiency of biological nitrogen fixation by improving legume-rhizobium symbiotic relationships is one of the most efficient ways, as it has a lower cost and smaller environmental impact, to increase the productivity of legumes, including soybean and to continue sustainable agriculture [3].

Soybean plants establish symbiotic relationships with soil rhizobia to fix atmospheric nitrogen. The establishment of such relationships involves complex mechanisms starting with the secretion by the host plant roots of particular chemical signaling compounds, mainly isoflavonoids, that are recognized by a compatible rhizobium and that induce the *nod* genes in that rhizobium [4]. In response, the rhizobium produces *Nod* factors, which are recognized by receptor kinases on plant roots, and these are responsible for initiating nodule formation in plants [5]. The principal signals, originating from the host plant and perceived by rhizobia in the soil, are luteolin, daidzein and genistein [6]. The isoflavonoids genistein and daidzein in soybean have been shown to be the primary inducers of *nod* gene expression in *Bradyrhizobium japonicum* [7–9]. Soybean isoflavonoids secreted in plant roots have been shown to induce *nod* gene expression in a specific rhizobium leading to nodulation [10]. Genistein alters the composition and molecular mass distribution of extracellular polysaccharides produced by *Rhizobium fredii* USDA193 [11].

Nodulation is a strictly controlled process because it not only consumes energy but also requires the tight control of a bacterial invader [12]. Rj(s) or rj(s) genes have been identified as controlling nodulation traits upon inoculation with compatible *Bradyrhizobium* and *Ensifer/Sinorhizobium* species. Among them, *Rj4*, found in cultivars Hill, Dunfield, Amsoy 71, Akisengoku and Fukuyutaka, induces inefficient nodulation with strains *B*. *japonicum* Is-34 and *B*. *elkanii* USDA61. Rj2/*Rfg1* gene restricts nodulation with the fast-growing *S*. *fredii* strains USDA257 and USDA205 [13]. A wide variety of bacterial strains with considerable diversity can nodulate with soybean plants in nature. For instance, *B*. *japonicum* USDA123 is predominant in soil from the northern USA, whereas *B*. *elkanii* USDA46, USDA76 and USDA94 are predominant in the southern USA [14]. *Sinorhizobium fredii* USDA205 and CCBAU114 and *Sinorhizobium xinjiangensis* CCBAU105 are the most effective strains in Brazilian field soil [15]. The representative clusters of the isolated bradyrhizobia change from *B*. *japonicum* strains USDA123, USDA110, and USDA6T to *B*. *elkanii* strain USDA76T as one moves from north to south within Japan [16]. Because of the variety of strains involved, it is yet not fully understood how the different indigenous rhizobial strains interact with all soybean genotypes or what genetic factors control their compatibility and affinity with a specific soybean cultivar.

Although many scientific works have underlined the connection between nodule rhizobia and isoflavonoids, none of them have been able to provide detailed genetic information about this relationship. Understanding this genetic interrelationship is important for developing strategies to improve the agronomic potential of root nodule symbiosis in agriculture. The current study sought to identify the genetic factors regulating soybean-rhizobium compatibility in the natural bacterial population and to determine the involvement of isoflavonoids in this compatibility using 93 RILs originating from Peking/Tamahomare (PT-RILs). For that purpose, we first identified the main indigenous rhizobia that have affinity with the parental cultivar lines Peking and Tamahomare by PCR-RFLP analysis targeted to the 16S-23S rRNA gene ITS region of the bacterial type of each root nodule. We then performed composite interval mapping analyses from three independent experiments to identify the QTLs controlling soybean-rhizobium compatibility. Finally, we cross-checked those QTLs with analyses of isoflavonoids from roots of 93 PT-RIL young seedlings and found that genestein is related to the genetic factors controlling soybean-rhizobium compatibility.

## Materials and methods

### Plant materials

We carried out two sets of experiments with 93 soybean (*Glycine max*) RILs from Tamahomare and Peking cultivars which have high compatibility with *B*. *japonicum* and *B*. *elkanii* respectively. Tamahomare is a Japanese cultivar with high yield but low protein content [17]. Peking is the Chinese landrace which has been extensively used as a genetic resource in soybean breeding and exhibits resistance to many nematodes and plant diseases [18–21].

### Bacterial nodule sampling

We performed three independent experiments. In the first two experiments, which were conducted in a greenhouse, we sowed six three-day pre-germinated soybean seeds of each line in a pot (10 cm x 20 cm x 15 cm) filled with field soil (90%) mixed with S sized pumice Kanuma soil (Tachikawa-heiwa Nouen Co., Ltd. Japan) (10%), which was commercially collected from the Kanuma pumice bed in the Kanuma area in Japan, to improve the soil structure. We took the field soil from a Kyoto University experimental farm field rotated between soybean fields and paddy fields every year. For these first (Exp1) and second (Exp2) experiments, the field soils were sampled after harvest of rice and soybean, respectively. Prior to the experiments, the soils were air dried and sieved (<5 mm sieve size). The seedlings were watered every two days and thinned to four plants on two weeks after sowing. We harvested the two best seedlings of each line at the fourth trifoliolate stage.

The third experiment (Exp3) was carried out in an incubator set at 28°C with a 18/6 h photoperiod using the method described by Shiro and colleagues [14] with some modifications. Soybean seeds were sterilized by being soaked in 70% ethanol for 30 s and in a 2.5% sodium hypochlorite solution for 3 min. They were rinsed with sterile distilled water and air dried. They were sown in culture pots filled with autoclaved (121°C for 20 min) vermiculite. Soybean seeds along with 2 g of soil from the continuous soybean cropping field (cultivar: Tambaguro) were placed in the vermiculite at a depth of 1 to 1.5 cm. The soil from the continuous soybean cropping field was provided by the Biotechnology Research Department, Kyoto Prefectural Agriculture, Forestry, and Fisheries Technology Research Center. The seedlings were moisturized daily with sterile distilled water for the first week and with Sterile N-free nutrient solution subsequently. We harvested the seedlings at the fourth trifoliolate stage. In all three experiments, we sampled 12 nodules per plant from two seedlings of each RIL and four seedlings from each of the Peking and Tamahomare cultivars.

### Bacterial DNA analysis from nodules and PCR-RFLP

The nodules were washed thoroughly to remove soil, surface sterilized with 70% ethanol for 1 min and 2.5% sodium hypochlorite solution for 3 min and rinsed three times with sterilized distilled water. They were transferred into a microplate (1 nodule/well) and crushed. After adding 10–25 μL TE buffer and 0,5–1 μL RNase to each well depending on nodule size, we heated the microplate at 99°C for 40 min. We added 5–10 μL bacterial lyse buffer and 1–2 μL Proteinase K, and boiled the microplate again at 99°C for 40 min. The samples were diluted 5 to 10 times with TE buffer for DNA templates.

0.5 μL DNA templates were mixed with 5 μL EmeralAmp Max PCR Master, 0.5 μL DMSO, 2.5 μL sterilized distilled water and 1.5 μL ITS primer set for PCR analysis [22]. The ITS primer sets used for the amplification of the 16S−23S rDNA ITS region of bradyrhizobia for the PCR reaction were: (BraITS-F: 5’-GACTGGGGTGAAGTCGTAAC -3’, BraITS-R: 5’-ACGTCCTTCATCGCCTC -3’); (ITS1512-F: 5’-GTCGTAACAAGGTAGCCGT-3’, ITSLS23-R: 5’-TGCCAAGGCATCCACC-3’); and (ITS320-F: 5’-TGGGGTGAAGTCGTAA-3’, ITS320-R: 5’-GGCCTGGGAAGACTTGAACT-3’). The PCR cycle consisted of a pre-run at 96°C for 2 min, denaturation at 96°C for 1 min, annealing at 55°C for 1 min and extension at 72°C for 2 min 30s. This was repeated for a total of 35 cycles and was followed by a final post-run at 72°C for 5 min.

For PCR-RFLP analysis of the 16S−23S rDNA ITS region, amplicons were digested with restriction enzyme *Hae*III, *Hha*I and *Xsp*I (TaKaRa Bio, Japan). Five microliters of each PCR product were digested with the restriction enzyme at 37°C for 6 h in a 10 μL reaction. The restricted fragments were electrophorased using submerged gel and stained with 1.5% ethidium bromide.

### Extraction of isoflavonoids

Seeds of the 93 soybean RILs from Tamahomare and Peking were surface sterilized with 70% ethanol for 1 min, then 2.5% sodium hypochlorite solution for 3 min, followed by four washes with sterilized distilled water. Surface sterilized seeds were sown in vermiculite-containing water and grown for six days at 25°C in the dark. Seedlings were removed, rinsed thoroughly and transferred into a hydroponic culture system containing 0.48 mM MgSO_4_, 1.2 mM KNO_3_, 0.168 mM KCl, 0.26 mM KH_2_PO_4_, 0.48 mM Ca(NO_3_)_2_, 4 μM Fe-EDTA, 9 μM KI, 52 μM MnCl_2_, 18 μM H_3_BO_3_, 4.6 μM ZnSO_4_, 1 μM CuSO_4_, and 0.006 μM Na_2_MoO_4_, pH 6.0, in 1 L of solution. The soybeans were grown in an incubator at 28°C with a 16/8 h photoperiod for 48h and isoflavonoids secreted into the hydroponic medium were collected.

Nutrient solution containing root exudates was filtered through Omnipore membrane filters (Millipore, Darmstadt, Germany), and its pH was adjusted to 3.0 using HCl. The medium was passed through a Sep-pak C18 Plus short cartridge (Waters, USA), which was washed with 3 mL water and eluted with 2 mL MeOH [23].

### HPLC analysis

HPLC analysis was conducted using a modification of the method described by Sugiyama and colleagues [23]. Isoflavonoids were analyzed with an HPLC machine (LC-10A, Shimadzu, Japan) under the following conditions: solvent A, 0.1% (v/v) acetic acid in filtered distilled water; solvent B, 0.1% (v/v) formic acid in acetonitrile; detection, 264 nm. Elution was at 0.8 mL/min with the solvent A (water containing 0.1% (v/v) acetic acid) and B (acetonitrile containing 0.1% (v/v) acetic acid) with a linear gradient program from 15 to 22% B in 40 min, followed by a linear gradient from 22 to 35% B in 40 min, and a linear gradient from 35 to 70% B in 5 min.

### QTL analysis

Phenotypic data analysis was conducted using SPSS Statistics 20.0 (SPSS, USA). One-way ANOVA for the analysis of each phenotype was performed.

We reconstructed the linkage map for 93 RILs (F_14_) using 227 polymorphic SSR (simple sequence repeat) markers with MAPMAKER/EXE v. 3.0 software [24] based on the linkage map for F_8_ plants consisting of 344 polymorphic SSR markers [25]. Quantitative trait locus mapping was performed by means of composite interval mapping (CIM) executed with WinQTL Cartographer 2.5 [26]. A QTL was declared significant when it had logarithm of odds ratio (LOD) threshold of 2.5 and permutation tests of 1000 times at a significance level of P = 0.05 [27]. Common QTL were verified for co-localization based on overlapping confidence intervals, defined as the range of a one-LOD drop on either side of the QTL peak.

## Results

### Nodule bacterial tendency of the parental lines and PT-RIL

**Fig 1:**
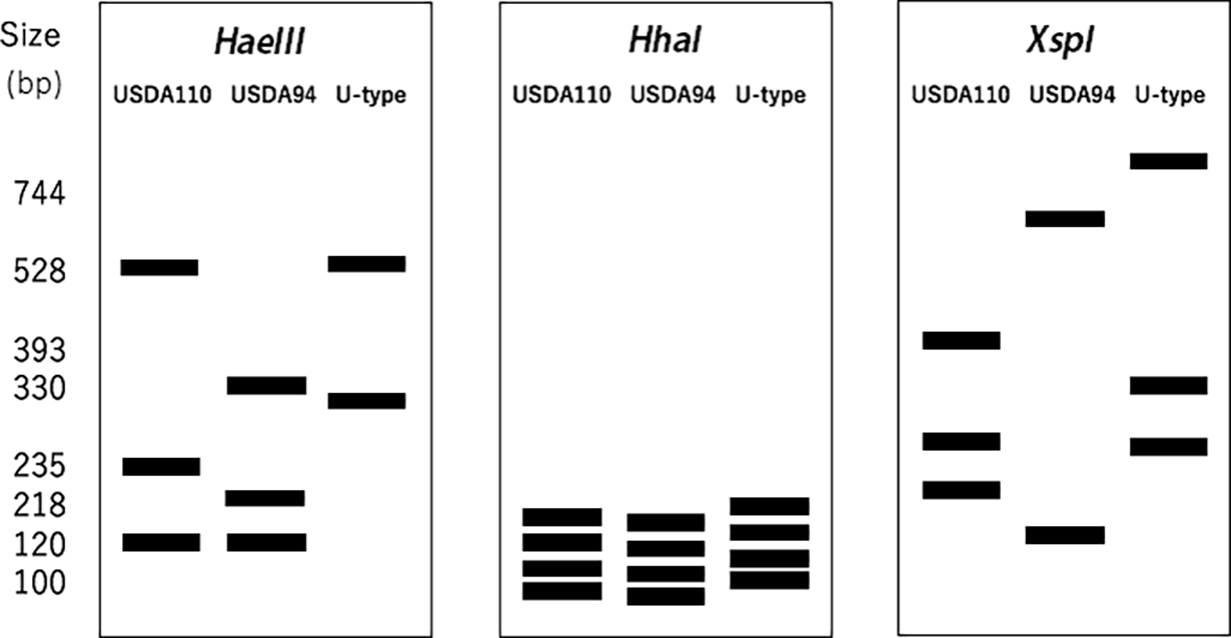
Schematic representation of amplicon patterns based on PCR-RFLP analysis of the l6S-23S rDNA internal transcribed spacer region of *B*. *japonicum* USDA110, *B*. *elkanii* USDA94, and *Bradyrhizobium sp*. (unidentified species).

We conducted PCR-RFLP analysis of the 16S−23S rDNA ITS region of bradyrhizobia in each nodule in order to identify the bacterial strains capable of forming nodules with Peking and Tamahomare cultivars. Based on the PCR-RFLP patterns of amplicons [28], we identified three groups of *Bradyrhizobium* strains: *B*. *japonicum* USDA110 (USDA110-type), *B*. *elkanii* USDA94 (USDA94-type) and other strains of *Bradyrhizobium sp*. (unidentified species, U-type) (Fig 1).

We carried out the same analysis for each of the 93 RIL. We compared the proportions of the bacterial types found in each nodule within and between genotypes and the three different field soil samples. The results indicated a highly significant difference in the proportions of USDA110-type, USDA110-type and U-type between genotypes (p<0.001). Peking was highly compatible with USDA94-type while Tamahomare was highly compatible with USDA110-type (Table 1). Peking did not nodulate with any other strain besides USDA-110 and USDA-94.

**Table 1:** Proportion (%) of bacterial types in nodules from Tamahomare, Peking and RILs from the three independent experiments.

Experiment	Tamahomare			Peking			Mean proportion of PT-RIL		
	USDA110-type	USDA94-type	U-type	USDA110-type	USDA94-type	U-type	USDA110-type	USDA94-type	U-type
Exp1	54.2	12.5	33.3	8.3	91.7	0	58.8	27.5	13.8
Exp2	41.7	45.8	12.5	4.2	95.8	0	55.5	38.0	6.1
Exp3	83.3	16.7	0	2.0	98.0	0	50.9	47.5	1.8

***Exp1***: seedlings cultivated in pot filled with soil from rice field (70%) mixed with pumice Kanuma soil (10%).

***Exp2***: seedlings cultivated in pot filled with soil from soybean field (70%) mixed with pumice Kanuma soil (10%).

***Exp3***: seedlings cultivated in pot set filled with vermiculite soil and 2 g of soil from the Tambaguro soybean field.

The proportions of USDA110-type bacteria found in association with Tamahomare and RIL were not significantly different between the experiments. The proportions of USDA94-type bacteria, however, were significantly higher (p<0.001) in Exp2 and Exp3 in all genotypes. Notably, the soil samples used in Experiments 2 and 3 were both from soybean fields, specifically, from the soil after a soybean harvest from rice-soybean rotation fields for Exp2 and from the continuous soybean cropping field for Exp3. In Exp3, the proportion of U-type was zero with Peking and Tamahomare and significantly lower than the proportions of the other types with RIL. Historical field cropping status might influence the properties of brarhizobia strains in the soil.

### QTL associated with compatibility of indigenous rhizobia with soybean

We detected 24 QTLs controlling the affinity of the indigenous USDA110-type, USDA94-type and unidentified U-type rhizobia strains with soybean in ten linkage groups (LG) (Table 2). The QTLs regulating the three nodule bacterial types were present in each experiment in different quantities. In Exp1, Exp2 and Exp3, we found eight, ten and six QTLs, respectively. In Exp3, 2 g of soil sampled from the Kyoto Prefectural experimental farm was mixed with vermiculite soil. In Exp1 and Exp2, soil samples (90%) taken from the Kyoto University experimental farm were mixed with Kanuma soil (10%). The lower number of identified QTLs in Exp3 was caused by the lower bacterial density in the soil sample used.

**Table 2:** Identified QTLs of *B*. *japonicum*, *B*. *elkanii*, *rhizobium sp*. in the PT-RIL population from three independent experiments.

Experiment	Trait	QTL	Chr (Linkage Group)	Nearest Marker	Peak position	LOD	Additive effect
Exp1	USDA110-type	qBJ_11	13 (F)	Satt335	122.2	3.65	9.30 ^t^
		qBJ_12	18 (G)	Sat_064	143.6	5.12	4.02^t^
	USDA94-type	qBE_11	13 (F)	Satt335	125.2	2.57	8.06 ^p^
		qBE_12	18 (G)	Sat_064	144.2	5.82	10.23 ^p^
	U-type-type	qBsp_11	2 (D1d)	Satt282	86.0	2.61	3.15 ^p^
		qBsp_12	18 (G)	Sat_064	145.0	6.12	4.82^t^
		qBsp_13	7 (M)	Satt150	9.4	3.17	4.39^t^
		qBsp_14	10 (O)	Sat_282	73.7	2.60	3.79^t^
Exp2	USDA110-type	qBJ_21	18 (G)	Sat_064	146.0	11.96	14.56 ^t^
		qBJ_22	20 (I)	SOYLBC_0	92.0	2.56	6.65 ^t^
		qBJ_23	3 (N)	Satt641	32.5	2.56	6.01 ^p^
	USDA94-type	qBE_21	14 (B2)	Satt577	0	4.11	8.95 ^p^
		qBE_22	2 (D1d)	Satt282	86.0	2.60	3.38 ^p^
		qBE_23	18 (G)	Sat_064	146.0	14.32	18.11 ^p^
		qBE_24	3 (N)	Satt641	32.5	3.43	7.79 ^t^
	U-type	qBsp_21	5 (A1)	Satt276	32.3	3.38	2.24 ^t^
		qBsp_22	2 (D1d)	Satt282	83.7	4.05	2.25 ^p^
		qBsp_23	18 (G)	Sat_064	144.0	11.47	3.56 ^t^
Exp3	USDA110-type	qBJ_31	3 (N)	Sct_195	5.2	2.83	12.10 ^p^
		qBJ_32	18 (G)	Sat_064, Sat_117	130.0	12.42	>35.5 ^t^
	USDA94-type	qBE_31	3 (N)	Sct_195	5.2	2.73	12.48 ^t^
		qBE_32	18 (G)	Sat_064, Sat_117	130.0	13.87	>35.5 ^p^
	U-type	qBsp_31	5 (A1)	Satt258	97.3	5.18	0.27 ^t^
		qBsp_32	11(B1)	Satt665	160.8	4.08	1.69 ^p^

***t*** Relative effect of Tamahomare-type allele compared with Peking-type allele.

***p*** Relative effect of Peking-type allele compared with Tamahomare-type allele.

In Exp1, two QTLs controlling symbiotic relation with USDA110-type (qBJ_11) and USDA94-type (qBE_11) were overlapped on Chr.13. In Exp1, the QTLs controlling symbiotic relation with USDA94-type (qBE_21) and U-type (qBsp_22) were located on Chr.2. At this same position we can find qBsp_11, which was shown to regulate symbiosis with U-type in Exp1. In Exp2 and Exp3, four QTLs controlling symbiotic relation with USDA110-type (qBJ_23 and qBJ_31) and USDA94-type (qBE_24 and qBE_31) coincided at almost the same position on Chr.3. These common QTLs were identified in both Exp2 and Exp3 because the soil samples used in both experiments had been taken from cultivated soybean fields. qBsp_21 of Exp2 and qBsp_31 of Exp3, both controlling symbiotic relation with U-type, were located at the same position on Chr.1. Likewise, qBsp_11 of Exp1 and qBsp_22 of Exp2 were found at the same position on Chr.2. QTLs located on Chr.18 were found in all three experimental conditions at almost the same position. Thus, the QTL located on Chr.18 should be derived from the significant genetic factor(s) that determine the compatibility of soybean plants with USDA110-type, USDA94-type and/or U-type bacterial species.

Overall, QTLs controlling symbiosis with the USDA110 type and the USDA94 type were often located at the same position as evidenced by the inversely correlated proportions of the USDA110 type and the USDA94 type. The effects of the Tamahomare-type allele increase compatibility with USDA110-type and decrease compatibility with USDA94-type.

### Isoflavonoid secretion from roots

We performed HPLC analysis of isoflavonoids of the root exudates of two plants per line in order to quantify the amount of daidzein and genistein secreted from each line. We did not detect glycitein secretion except in trace amounts from a few lines. Daidzein secretions from Peking and Tamahomare were 43.60μg and 14.24μg, respectively, while genistein secretions were 0.28μg and 0.73μg, respectively. Daidzein was secreted much more than genistein. Daidzein and genistein secretions from RIL plant roots range from 0.25 to 222.03 *μ*g and from 0.18 to 1.37 *μ*g, respectively. The mean amount of daidzein secreted from the RIL was significantly higher than that secreted from Peking or Tamahomare. The mean amount of genistein secreted from the RIL, however, was significantly higher than that secreted from Peking but not significantly different from that secreted from Tamahomare.

Six QTLs controlling isoflavonoid secretions of roots were identified on five different chromosomes (Table 3). Daidzein and genistein exudates were regulated according to chromosomal position. The QTL with the largest effect on genistein was detected on Chr.13, while that with the second largest effect on genistein was detected on Chr.18. At each of these QTLs, the allele associated with very low genistein secretion was derived from Tamahomare. The QTL with the largest effect on daidzein was detected on Chr.8. The alleles affecting daidzein on all chromosomes were derived were Tamahomare.

**Table 3:** Identified QTLs controlling daidzein and genistein secretions from roots detected by means of composite interval mapping in the PT-RIL population.

Trait	QTL	Chr (Linkage Group)	Nearest Marker	Peak position	LOD	Additive effect
Daidzein	qDZS1	8 (A2)	Sat_162	51.4	3.14	21.27 ^t^
	qDZS2	8 (A2)	Sat_377	106.8	2.66	18.30 ^t^
	qDZS3	10 (O)	Sat_282	70.5	2.86	20.28 ^t^
Genistein	qGNS1	6 (C2)	Satt520	38.2	2.55	0.07 ^t^
	qGNS2	13 (F)	Satt335	102.9	6.88	0.15 ^t^
	qGNS3	18 (G)	Sat_064	144.3	4.01	0.14 ^t^

***t*** Relative effect of Tamahomare-type allele compared with Peking-type allele.

### Interrelationship between isoflavonoid secretion of soybean and affinity of *B*. *japonicum* and *B*. *elkanii* with soybean

Our results revealed that the eight overlapping QTLs controlling the affinity of indigenous rhizobia strains with soybean, nearby Sat_064 on Chr.18, coincided with a QTL controlling genistein secretion from soybean roots (Table 2, Table 3). In other words, there is a genetic interrelationship between genistein and the compatibility of indigenous rhizobia strains with soybean. There was no correlation between daidzein and soybean compatibility with any nodulating bacteria strain (S2 Fig).

**Fig 2:**
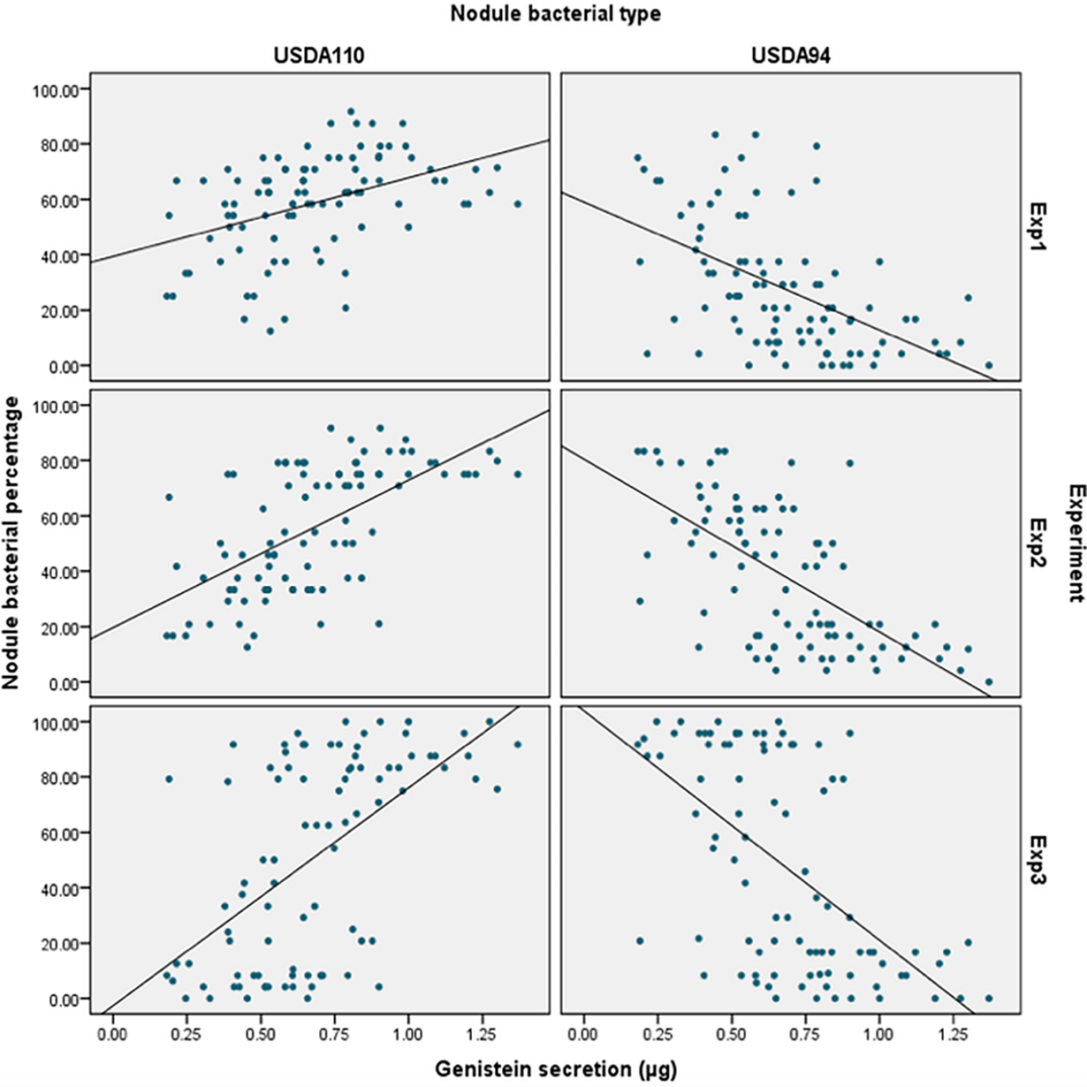
Relationship between genistein secretion and percentage of UDSA110-type and USDA94-type nodules from three independent experiments.

We compared the percentages of USDA110-type and UDSA94-type strains with levels of genistein secretion in order to confirm their relationship, and found that the genistein secretion trait was positively correlated with USDA110-type traits and negatively correlated with USDA94-type traits in all experiments (Fig 2). These correlations and regressions appeared to be stronger in Exp2 and Exp3, both conducted in soybean field soil, than they were in Exp1, conducted in rice field soil, suggesting that historical field cropping status influence the affinity of indigenous rhizobia strains with soybean.

## Discussion

Of the 24 QTLs that we identified, eight QTLs coincided at the same position on Chr.18 near Sat_064 in all three experimental conditions. Accordingly, this is regarded as the most relevant QTL region of our research. No previous report has mentioned this chromosomal region or its vicinity in connection with nodulation. Therefore, these alleles, which are derived from both Tamahomare and Peking varieties, are considered to be novel QTLs controlling the compatibility of indigenous rhizobia (USDA110-type, USDA94-type and/or U-type bacterial species) with soybean plant. Another four QTLs controlling symbiotic relation with the USDA110-type and USDA94-type were located at almost the same position on Chr.3 near Sct_195 and Satt641. Alleles at these QTLs that were derived from Tamahomare are associated with high symbiotic compatibility with USDA110-type bacteria, while those that were derived from Peking are associated with high symbiotic compatibility with USDA94-type bacteria.

Concerning isoflavonoids, our results revealed six QTLs controlling daidzein and genistein secretion levels from soybean roots. The current QTLs are in accordance with the results of previous measurements of isoflavonoid contents of soybean seeds. qDZS1 on Chr.8, qGNS1 on Chr.6 and qGNS3 on Chr.18 have been reported by Yoshikawa and colleagues [29], while qDZS3 and qGNS2 have been reported by Wang and colleagues [30]. This indicates that the isoflavonoid contents in seeds and their secretions from roots of soybean are regulated by the same genetic factors. Daidzein and genistein levels in seeds are not very different among Peking, Tamahomare and the RILs [29]. In the roots of RIL plants, however, we found that daidzein secretions were nearly 100 times greater than genistein secretions in the early stage of growth (seven days after sowing). Most isoflavonoids in root exudates in the early stages of soybean plant growth were daidzein derivatives as well [23]. There are large differences in the quantity of iso/flavonoid exudation at different positions along the root, with larger amounts reported to be exuded from the root tip [31]. The secretion of genistein from soybean roots involves an ATP-binding cassette-type transporter [32].

Amongst the QTLs of isoflavonoid traits, one that controls genistein secretion from soybean roots was located in the vicinity of Sat_064 on Chr.18. This region is also the most relevant chromosomal region related to the regulation of soybean-rhizobium compatibility as we highlighted above. This implies that genistein is one of the major factors controlling the compatibility of indigenous rhizobia strains with soybean. Daidzein and genistein have been known as *nod* gene expression inducers [7–9]. However, our results showed that the principal attractant for USDA110-type nodule bacteria was genistein, but the roles of daidzein were not observed. Genistein at a concentration of 1 μM changed exopolysaccharide concentration and composition in *Rhizobium fredii* cultures [11]. Also, several of the protein spots were detectable only after the addition of genistein to a *B*. *japonicum* culture [33]. Moreover, pre-incubation of the *B*. *japonicum* inoculant with genistein probably contributed to growth in soybean through enhancement of nodulation and nitrogen fixation and alleviation of salt and drought stresses [34–37]. Therefore, we suggest that genistein alone induces *nod gene* expression in *B*. *japonicum*.

The two other QTLs regulating genistein secretion from roots, qGNS1 and qGNS2, are located in a chromosomal position not related to the compatibility of indigenous rhizobia strains with soybean. The existence of these two QTLs indicates that genistein secretion from roots is controlled by more than one factor. Differences of QTLs among experimental conditions, Exp1, Exp2 and Exp3, suggest that isoflavone contents secreted from soybean root, especially genistein, are altered by environmental condition such as soil and field cropping history. Secreted genistein from root is strongly influenced by genotype and environment as well as isoflavones in seeds [29,30,38–40].

The chromosomal region in the vicinity of Sat_064 on Chr.18 is related to soybean cyst nematode resistance genes in Peking [41,42] according to the integrated map of GmComposite2003_G (www.soybase.org). The genes that restrict nodulation with specific rhizobial strains resemble those encoding plant-pathogen resistant proteins (R) because symbiosis incompatibility is controlled in a manner similar to gene-for-gene resistance against plant pathogens [43,44]. *Rj2* and *Rfg1* genes encode a typical Toll-interleukin receptor/nucleotide-binding site/leucine-rich repeat resistance protein that prevents nodulation with specific strains of *B*. *japonicum* and *S*. *fredii*, respectively [45]. *Rj4* encodes a thaumatin-like pathogenesis-related protein that restricts nodulation by specific strains of *B*. *elkanii* [46]. Besides, plant growth-promoting rhizobacteria not only support plant growth but also provide systemic protection against diseases [47]. Moreover, genistein is detrimental to some bacteria [48,49]. Isoflavonoids can act as inducers for certain rhizobia and anti-inducers (antagonists) for others [50]. The negative correlation between the USDA94-type and genistein suggests that the genistein could be an inhibitor of nod gene expression in *B*. *elkanii*.

The effects of the Tamahomare-type allele were stronger on QTLs involved in compatibility with the USDA110 type, whereas, inversely, the effects of the Peking-type allele were stronger on QTLs involved in compatibility with the USDA94 type. Since Tamahomare is a Japanese cultivar, other Japanese genotypes such as Tambaguro and Enrei may have the same compatibility tendencies as Tamahomare, i.e., greater compatibility with *B*. *japonicum* USDA110 [51]. Also, Japanese and Chinese genotypes may restrict nodulation with *B*. *japonicum* USDA6, USDA38, and USDA115, given that, in Kyoto, Japan, where the current research was carried out, *B*. *japonicum* USDA110 (45%) is the dominant strain in the field, followed by *B*. *japonicum* USDA6 (23.5%), USDA38 (13%), USDA115 (11.5%) and *B*. *elkanii* USDA94 (7%) [51], though we observed only the *B*. *japonicum* USDA110 and *B*. *elkanii* USDA94 strains in symbiosis with our soybean plants.

In conclusion, among the 24 QTLs identified in this study that regulate the compatibility of indigenous rhizobia strains with soybean, seven were located at the same chromosomal position along with a QTL controlling genistein secretion from soybean roots. Therefore, this result suggests that genistein plays a crucial role in forming nodule and adjusting the proportion of bacterial strains to nodulate. The current work is a first step toward an efficient way to investigate the genes regulating the symbiotic relationships of legumes with compatible bacterial strains.

## Supporting information

S1 Fig RFLP patterns of the 16S-23S rRNA gene ITS region of the three nodule bacterial types identified from Peking and Tamahomare. ***HaeIII*), *HhaI*)**, and ***XspI*)** represent the results from restriction enzymes *HaeIII*, *HhaI* and *XspI*, respectively. ***a***, ***b*** and ***c*** represent *B*. *japonicum* USDA110, *B*. *elkanii* USDA94, and *Bradyrhizobium sp*., respectively.
(TIF)

S2 Fig Correlation between daidzein and percentage of UDSA110- and USDA94-type nodules from three independent experiments. (TIF)

S1 File Whole Data. ***Parents***. Number and percentage of bacterial types in 48 nodules (12 nodules/plant) found on Peking and Tamahomare plants from three independent experiments. Figures show the PCR-RFLP patterns of nodule bacterial types out of 48 nodules on Peking and Tamahomare plants from three independent experiments. Statistical analysis and one-way ANOVA between plants and experiments using *B*. *japonicum* USDA110, *B*. *elkanii* USDA94 and *Bradyrhizobium sp*. ***RIL Exp1***: Number and percentage of nodule bacterial types of RIL from Exp1. ***RIL Exp2***: Number and percentage of nodule bacterial types of RIL from Exp2. ***RIL Exp3***: Number and percentage of nodule bacterial types of RIL from Exp3. ***3exp summary***: Summary of the percentage of nodule bacterial types of RIL (mean value, max, min, frequencies). Statistical analysis and one-way ANOVA between experiments of *B*. *japonicum* USDA110, *B*. *elkanii* USDA94 and *Bradyrhizobium sp*. ***Isoflavonoid***: Daidzein and genistein secretion from soybean roots of RIL, Peking and Tamahomare. Statistical analysis and one-way ANOVA between the two replicated plants and between RIL, Peking and Tamahomare. ***QTL***: Figures show the 22 QTLs identified on six chromosomes. ***Correlation***: Figures show the correlations between isoflavonoid secretion from soybean roots and compatibility with USDA110 and USDA94.
(XLSX)
