# Supplementary figures and images for: QTLs underlying the genetic interrelationship between efficient compatibility of Bradyrhizobium strains with soybean and genistein secretion by soybean roots
Source: PLoS One. 2018 Apr 4;13(4):e0194671. doi: 10.1371/journal.pone.0194671 (PMC5884529; doi:10.1371/journal.pone.0194671)

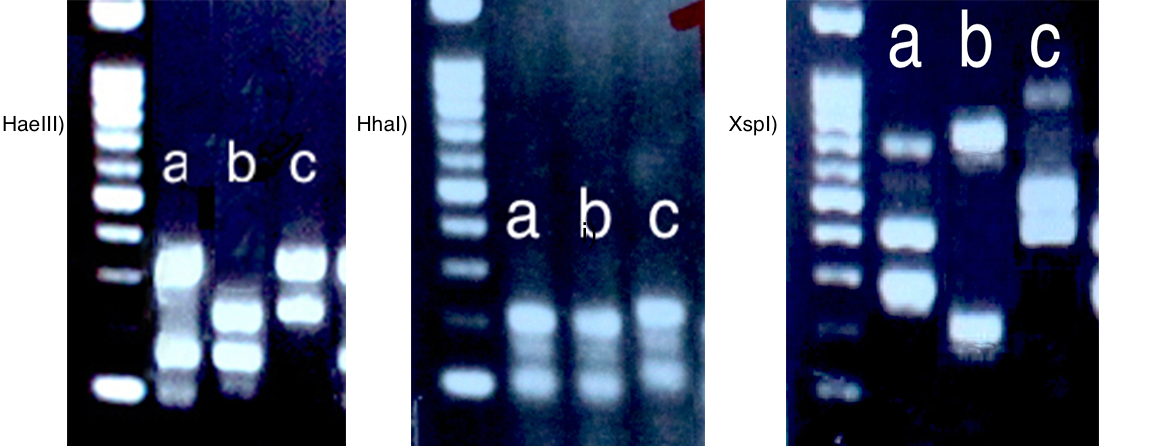

Supplement: S1 Fig — HaeIII), HhaI), and XspI) represent the results from restriction enzymes HaeIII, HhaI and XspI, respectively. a, b and c represent B. japonicum USDA110, B. elkanii USDA94, and Bradyrhizobium sp., respectively. (TIF) [file pone.0194671.s001.tif]

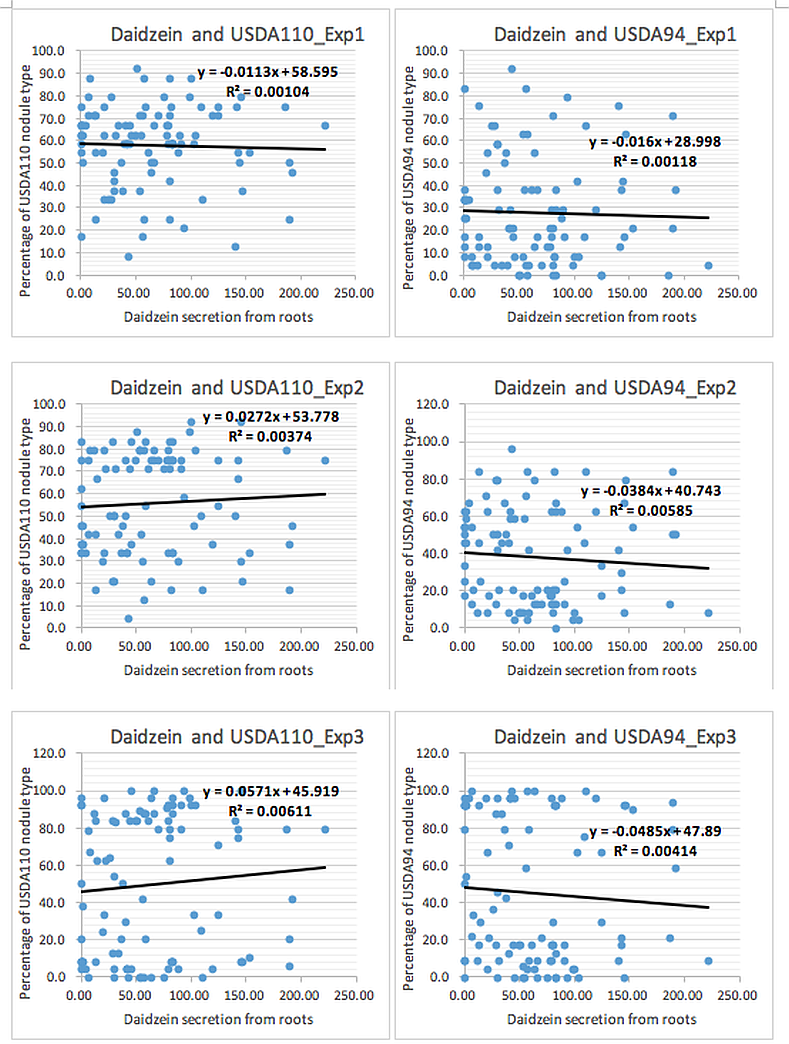

Supplement: S2 Fig — (TIF) [file pone.0194671.s002.tif]
